# Advances in pharmacologic management of obesity: oral semaglutide for weight management

**DOI:** 10.1097/MS9.0000000000005161

**Published:** 2026-09-08

**Authors:** Fnu Zainab, Abdul Haseeb Hasan

**Affiliations:** a Department of Medicine, King Edward Medical University, Lahore, Pakistan; b Department of Research, Oli Health Magazine Organization, Kigali, Rwanda; c Department of Internal Medicine, Sentara Northern Virginia Medical Center, Woodbridge, VA, USA

**Keywords:** FDA, GLP-1, OASIS trial, obesity, semaglutide, Wegovy

## Abstract

Obesity is a chronic, multifactorial disease characterized by excessive adiposity and associated with an increased risk of cardiovascular disease, type 2 diabetes, and reduced quality of life. Despite the widespread adoption of lifestyle interventions, long-term weight maintenance remains challenging, highlighting the need for effective pharmacologic therapies. Recent advances, including the US Food and Drug Administration approval of oral semaglutide, offer new opportunities for patient-centered obesity management. Evidence from the OASIS 1 and OASIS 4 trials demonstrates that oral semaglutide, combined with lifestyle intervention, produces clinically meaningful weight loss in adults with overweight or obesity without type 2 diabetes, while improving physical function and cardiometabolic risk markers, such as waist circumference, lipids, and inflammatory parameters. These trials further underscore the benefits of an oral formulation in improving accessibility and adherence compared with injectable therapies. Collectively, oral semaglutide represents a significant advancement in obesity pharmacotherapy, complementing lifestyle interventions and providing both metabolic and functional benefits. Future studies addressing long-term efficacy, cardiovascular outcomes, comparative effectiveness with other agents, and real-world implementation will be essential to fully define its role in comprehensive, individualized obesity care.

## Introduction

Obesity and/or overweight are characterized by an excessive or abnormal accumulation of body fat that can negatively impact health. Body mass index (BMI), calculated as a person’s weight in kilograms divided by the square of their height in meters (kg/m^2^), is commonly used to assess this risk. According to the World Health Organization (WHO), adults with a BMI of 25 or higher are considered overweight, while a BMI of 30 or higher is classified as obese^[^1^]^. Excess body weight, whether overweight or obesity, can have serious health implications, increasing the risk of cardiovascular diseases such as heart disease and stroke, musculoskeletal disorders including osteoarthritis, type 2 diabetes, and certain cancers, notably breast, endometrial, and colorectal cancers^[^2^]^. About 2.5 billion adults were overweight in 2022, with over 890 million of those persons being obese^[^3^]^. Overweight and obesity combined affected more than 2 billion adults worldwide by 2021, a dramatic rise from 731 million in 1990, and this burden is expected to grow further without systemic intervention^[^3^]^. Obesity has now achieved epidemic proportions globally, with at least 2.8 million people dying each year as a result of being overweight and obese^[^2^]^.

HIGHLIGHTS

- Oral semaglutide is the first oral GLP-1 receptor agonist approved for chronic weight management in adults with overweight or obesity.
- In the OASIS-1 trial, oral semaglutide 50 mg achieved a mean weight reduction of 15.1% compared with 2.4% with placebo.
- The OASIS-4 trial demonstrated significant weight loss with oral semaglutide 25 mg and improved physical function compared with placebo.
- Oral semaglutide was associated with favorable changes in waist circumference, lipid profiles, and inflammatory markers.
- Oral GLP-1 therapy may improve treatment accessibility and adherence while providing an effective alternative to injectable anti-obesity medications.

## Causes and risk factors of obesity

Obesity is a multifactorial condition resulting from the interaction of biological, behavioral, environmental, and social determinants rather than individual choice alone. Non-modifiable factors, such as genetic predisposition, interact with modifiable influences, including excess caloric intake, physical inactivity, inadequate sleep, psychosocial stress, and early-life exposures^[^4^]^. Personal habits of an individual can significantly affect health. Genetic and epigenetic factors play a significant role in obesity susceptibility, with evidence from genome-wide association studies identifying numerous genetic variants associated with BMI and adiposity. These variants primarily influence biological pathways related to appetite regulation, energy balance, lipid metabolism, and fat storage^[^5^]^. Beyond inherited genetic variation, epigenetic mechanisms, particularly DNA methylation, link environmental and lifestyle exposures, such as diet, physical activity, and smoking, to long-term changes in gene expression that promote obesity^[^5^]^. Exposure to endocrine-disrupting chemicals (EDCs), also known as obesogens, has been implicated as a contributing factor to obesity through the disruption of hormonal pathways regulating metabolism and adipogenesis^[^6^]^. These chemicals, commonly present in plastics, pesticides, and consumer products, may increase fat accumulation and obesity risk, particularly when exposure occurs during early developmental periods. Their ability to accumulate in adipose tissue may further exacerbate metabolic dysfunction and weight gain^[^6^]^. Additional contributors include certain medications, underlying medical conditions, socioeconomic status, ethnicity, EDCs, and alterations in the gut microbiome, all of which operate within an increasingly obesogenic environment^[^4^]^. As a result, obesity prevention and management require comprehensive, system-level approaches rather than solely focusing on personal behavior changes.

## Non-pharmacological and pharmacological options for the treatment of obesity

Obesity should be managed like other chronic diseases, as it is a progressive condition with a high risk of recurrence. Maintaining a healthy weight can be supported by a balanced diet that emphasizes fruits, vegetables, pulses, whole grains, and nuts; limits added sugars and salt; and reduces total fat intake, particularly by replacing saturated fats with unsaturated fats^[^2^]^. Regular physical activity also plays a key role in maintaining a healthy weight, with at least 150 minutes of moderate-intensity exercise per week shown to significantly reduce obesity-related health risks^[^7^]^. The US Food and Drug Administration (FDA) has approved several pharmacological therapies for obesity, with many additional medications used off-label. Current medications approved by the FDA for the treatment of obesity are divided into two groups: first-generation (Table 1) and second-generation drugs (Table 2), with each group having limitations in terms of ideal candidates, adverse effects, and contraindications^[^8^]^.

**Table 1:** First generation of FDA-approved drugs for obesity.

Drug	Class	Key clinical benefits
Bupropion	A dopamine and norepinephrine reuptake inhibitor	Reduces appetite by increasing the activity of pro-opiomelanocortin neurons in the hypothalamus^[^9^]^.
Naltrexone	An opioid receptor antagonist	Blocks inhibitory feedback on pro-opiomelanocortin neurons^[^10^]^.
Phentermine	Sympathomimetics	Stimulate the release of norepinephrine, leading to decreased appetite and reducing hunger^[^11^]^.
Diethylpropion		
Topiramate extended release	An antiepileptic agent	Exerts anorexic effect via modulation of gamma-aminobutyric acid receptors in the brain^[^12^]^.
Metformin	A biguanide	Influences central appetite-regulatory pathways, including effects on hypothalamic signaling, and increases the release of gut hormones that suppress hunger (e.g., GLP-1 and peptide YY)^[^13^]^.
Orlistat	Lipase inhibitor	Acts locally in the gut to reduce fat absorption^[^14^]^.

**Table 2:** Second generation of FDA-approved drugs for obesity.

Drug	Class	Key clinical benefits
Liraglutide and semaglutide	GLP-1 receptor agonists	Significantly promote weight loss in people with overweight or obesity compared with placebo, with semaglutide generally producing greater weight reduction than liraglutide^[^18^]^.
Tirzepatide	A dual glucose-dependent insulinotropic polypeptide (GIP) and glucagon-like peptide-1 (GLP-1) receptor agonist	Produces significant reductions in body weight compared with placebo and other comparators in adults with type 2 diabetes mellitus and obesity^[^19^]^.

## First generation

A combination of extended-release bupropion with extended-release naltrexone is approved by the FDA for the treatment of chronic obesity^[^15^]^. A combination of phentermine with topiramate extended-release exerts a greater weight loss effect than either of these used alone, and it is approved by the FDA for weight loss^[^16^]^.

For individuals who are overweight or obese (BMI ≥ 30 kg/m^2^, or ≥ 27 kg/m^2^ with weight-related comorbidities) and do not achieve sufficient results with lifestyle interventions alone, combining pharmacologic treatment with lifestyle modifications is recommended. The American Gastroenterological Association (AGA) guideline panel provided nine recommendations for obesity management. For long-term treatment, they advised using semaglutide 2.4 mg, liraglutide 3.0 mg, phentermine-topiramate ER, and naltrexone-bupropion ER (supported by moderate-certainty evidence), as well as phentermine and diethylpropion (supported by low-certainty evidence). The panel recommended against the use of orlistat for long-term therapy^[^17^]^.

## Second generation

Second-generation medications for weight loss mimic incretins that act on glucagon-like peptide (GLP-1) and glucose-dependent insulinotropic polypeptide (GIP) receptors, leading to delayed gastric emptying, increased satiety, and reduced appetite to promote weight loss. FDA-approved second-generation medications for the long-term treatment of obesity are shown in Table 2.

## Emerging oral GLP-1 agonist for weight loss

Recently, the FDA has approved an oral GLP-1 agonist, Wegovy, a semaglutide, for the treatment of obesity^[^20^]^. The approval was based on data from a randomized, double-blind, placebo-controlled phase 3 OASIS-1 trial^[^21^]^ and phase 3 OASIS-4 trial^[^22^]^.

## OASIS-1 trial

The OASIS-1 trial was a phase 3, randomized, double-blind, placebo-controlled study that included adults with overweight (BMI ≥ 27 kg/m^2^) or obesity (BMI ≥ 30 kg/m^2^) without type 2 diabetes. A total of 709 participants were randomly assigned in a 1:1 ratio to receive oral semaglutide 50 mg once daily (*n* = 334) or placebo (*n* = 333), combined with lifestyle interventions (diet and exercise) over 68 weeks^[^23^]^. The primary endpoint was the percentage change in body weight from baseline. Participants treated with oral semaglutide achieved a mean weight reduction of 15.1% versus 2.4% with placebo, and significantly more participants reached weight-loss thresholds of ≥5%, ≥10%, ≥15%, and ≥20%^[^21^]^.

## OASIS-4 trial

The clinical trial included 307 adults with obesity (BMI ≥ 30 kg/m^2^) or overweight (BMI 27–29.9 kg/m^2^) and at least one weight-related comorbidity, without diabetes. The mean baseline body weight was 106 kg, with a BMI of 38 kg/m^2^. Participants either received semaglutide 25 mg once daily or a placebo once daily. This study lasted for 72 weeks, which included a 1-week screening period, 64 weeks of treatment, and 7 weeks of follow-up^[^22^]^. Participants were randomly assigned in a 2:1 ratio to receive oral semaglutide 25 mg (*n* = 205) or placebo (*n* = 102) once daily, along with lifestyle modifications such as a reduced-calorie diet and increased physical activity for a period of 64 weeks. Participants receiving oral semaglutide experienced an average weight reduction of 13.6% from baseline to week 64, compared with 2.2% in the placebo group, resulting in an estimated difference of 11.4 percentage points [95% confidence interval (CI), −13.9 to −9.0; *P* < 0.001)]. Those on oral semaglutide also showed significant improvements in physical function (*P* < 0.001) and were much more likely to achieve weight loss of 5%, 10%, 15%, or 20% compared with placebo (*P* < 0.001 for all thresholds)^[^22^]^. Oral semaglutide caused higher gastrointestinal side effects than placebo (74.0% vs. 42.2%)^[^24^]^.

OASIS 4 trial underscores that oral semaglutide 25 mg not only produces significant weight loss comparable to higher oral doses (50 mg in OASIS 1) and the injectable semaglutide 2.4 mg, but also provides meaningful metabolic and functional benefits^[^25^]^. Participants experienced improvements in cardiometabolic risk factors, including reductions in waist circumference, lipids, and inflammatory markers such as C-reactive protein, which may translate into long-term cardiovascular risk reduction^[^25^]^. Additionally, physical function and quality-of-life measures improved, indicating that weight loss was accompanied by enhanced mobility and daily functioning. By demonstrating both efficacy and broader health benefits, OASIS 4^[^22^]^ highlights the potential of oral semaglutide as a versatile and patient-centered option in obesity management, suggesting that pharmacologic therapy can simultaneously target weight, metabolic health, and functional outcomes.

## Limitations

Both the OASIS-1 and OASIS-4 randomized, double-blind, placebo-controlled trials evaluated the efficacy and safety of oral semaglutide combined with standardized lifestyle intervention in adults with overweight or obesity without type 2 diabetes, demonstrating clinically meaningful weight loss versus placebo^[^22,23^]^. However, important limitations warrant consideration. The trials used placebo comparators rather than active weight-loss agents, limiting direct insight into how oral semaglutide compares with contemporary pharmacologic alternatives. Both studies applied stringent inclusion and exclusion criteria and were conducted in select outpatient settings, which may reduce generalizability to more diverse real-world populations, particularly across wider age ranges, sociodemographic groups, and patients with significant comorbidities not represented in the cohorts. The reliance on standardized lifestyle modifications may not fully reflect routine clinical practice, and adherence to behavioral interventions may differ outside a controlled trial environment. While OASIS-1 was a large phase 3 study with multiple international sites, OASIS-4 enrolled a relatively smaller sample, which could limit statistical power for certain secondary outcomes and subgroup analyses^[^22^]^. Additionally, both trials’ durations (up to ~68 weeks) constrain long-term assessment of sustained weight maintenance, cardiovascular outcomes, and rare or delayed adverse events.

Furthermore, the controlled trial environment may overestimate medication adherence compared to real-world settings, particularly given the strict administration requirements of oral semaglutide, including fasting intake and delayed food consumption. Real-world data demonstrate substantially lower persistence and adherence rates compared to clinical trials, which may reduce observed effectiveness despite strong efficacy signals in randomized settings^[^26^]^. In addition, adherence patterns are influenced by a complex interplay of patient-specific factors, including comorbidities, treatment burden, and patient preferences, which are not fully captured in controlled trial environments^[^27^]^. Another important limitation is the absence of direct head-to-head comparisons with other established anti-obesity pharmacotherapies. This is particularly relevant given real-world evidence demonstrating substantial interindividual variability in response to oral semaglutide, with a significant proportion of patients failing to achieve clinically meaningful weight loss and overall efficacy appearing lower than injectable GLP-1 receptor agonists^[^28^]^. These findings further highlight a translational gap between clinical trial efficacy and real-world effectiveness, where outcomes may be attenuated due to variability in patient response and adherence outside controlled settings^[^28^]^. Moreover, semaglutide-induced weight loss is primarily mediated through reduced energy intake, which may vary significantly across individuals, further contributing to heterogeneity in clinical response and treatment outcomes^[^29^]^.

## Future perspective

The OASIS-1 and OASIS-4 trials have demonstrated that oral semaglutide, when combined with lifestyle intervention, can achieve clinically meaningful weight reductions in adults with overweight or obesity without type 2 diabetes, expanding the potential therapeutic landscape beyond injectable GLP-1 receptor agonists. OASIS-1 showed substantial mean weight loss with a 50 mg dose over 68 weeks^[^23^]^, while OASIS-4 confirmed significant weight loss with a 25 mg dose over 64 weeks, including improvements in physical function and quality-of-life measures compared with placebo^[^22^]^. Looking forward, these trials pave the way for several key advances in anti-obesity pharmacotherapy. Oral formulations may increase patient adherence and accessibility, particularly among individuals averse to injections or those with limited access to specialized care – issues that have historically constrained the uptake of injectable therapies. Furthermore, the demonstration of significant weight loss comparable to established injectable agents suggests that oral semaglutide could broaden the standard of care if future research confirms similar cardiometabolic and long-term health benefits^[^25^]^.

Recent advances in artificial intelligence, particularly large language models such as ChatGPT, have introduced new opportunities in the management of chronic metabolic disorders. A recent global-scale bibliometric analysis demonstrated a rapid expansion of ChatGPT applications in medicine while highlighting key concerns related to accuracy, safety, and ethical considerations^[^30^]^. In the context of oral semaglutide therapy, these tools may play a supportive role in improving patient education, particularly regarding its strict administration requirements and potential adverse effects. Furthermore, ChatGPT-based systems could enhance clinical decision support by assisting clinicians in selecting appropriate candidates for GLP-1 receptor agonist therapy and optimizing dose titration. However, the same analysis emphasizes that clinical decision-making applications remain underdeveloped and require cautious integration into practice^[^30^]^. Importantly, concerns regarding misinformation and patient safety are highly relevant in chronic diseases such as type 2 diabetes, where inappropriate guidance may adversely affect outcomes. Additionally, disparities in access to AI technologies may further widen global healthcare inequalities, particularly in resource-limited settings where oral semaglutide could otherwise offer a convenient therapeutic option^[^30^]^. Therefore, while ChatGPT holds promise in enhancing pharmacotherapy, its integration into clinical care must remain supervised, ethical, and evidence-based.

To fully realize this potential, future studies should aim to address current limitations. Long-term trials extending beyond 68 weeks are needed to evaluate the durability of weight maintenance, impacts on cardiovascular outcomes, and rare or delayed adverse events. Comparative studies against other active pharmacologic treatments rather than placebo would clarify relative efficacy and cost-effectiveness, while inclusion of more diverse and comorbid patient populations would enhance generalizability and inform guideline-based care. Additionally, real-world effectiveness research and implementation studies could elucidate how lifestyle intervention adherence and patient behavior interact with pharmacotherapy outside controlled trial settings. Together, these advancements would solidify the role of oral semaglutide and other oral GLP-1 agents in the evolving paradigm of obesity management and support personalized, accessible treatment strategies for a broad spectrum of patients.

## Conclusion

Obesity remains a major global health challenge, driven by complex interactions among genetic, metabolic, behavioral, and environmental factors, and is associated with significant cardiometabolic and functional risks. While lifestyle interventions, including diet, exercise, and behavioral strategies, remain the cornerstone of management, sustained weight loss is often difficult to achieve, highlighting the need for effective pharmacologic options. Recent advances, particularly the FDA approval of oral semaglutide (Wegovy^®^) for chronic weight management, represent a significant step forward in obesity treatment. The OASIS-1 and OASIS-4 trials provide robust evidence supporting the efficacy and safety of oral semaglutide in adults without type 2 diabetes, demonstrating not only clinically meaningful weight loss but also improvements in physical function and cardiometabolic risk markers, with a tolerable safety profile. These findings suggest that oral semaglutide may expand access to effective pharmacotherapy, complement lifestyle interventions, and could play a central role in personalized, patient-centered obesity management. Future studies addressing long-term outcomes, real-world effectiveness, and comparative efficacy with other pharmacologic agents will further define its role in comprehensive obesity care.

## Data Availability

Not applicable.
